# Informative High-Risk HPV Genotyping in Cervical Cancer Screening: Integrated Analysis of Cytology and p16/Ki67 Dual Staining

**DOI:** 10.3390/cancers18071056

**Published:** 2026-03-25

**Authors:** Martyna Trzeszcz, Karolina Mazurec, Maciej Mazurec, Christopher Kobierzycki, Agnieszka Halon, Robert Jach

**Affiliations:** 1 Division of Pathology and Clinical Cytology, University Hospital in Wroclaw, 50-556 Wroclaw, Poland; karolina.mazurec@umw.edu.pl; 2 Corfamed Woman’s Health Center, 50-322 Wroclaw, Poland; m.mazurec@corfamed.pl; 3 Division of Clinical Pathology, Department of Clinical and Experimental Pathology, Wroclaw Medical University, 50-556 Wroclaw, Poland; agnieszka.halon@umw.edu.pl; 4 Division of Histology and Embryology, Department of Human Morphology and Embryology, Wroclaw Medical University, 50-368 Wroclaw, Poland; christopher.kobierzycki@umw.edu.pl; 5 Division of Gynecologic Endocrinology, Jagiellonian University Medical College, 31-501 Krakow, Poland; jach@cm-uj.krakow.pl

**Keywords:** human papillomavirus (HPV), HPV genotyping, extended genotyping, limited genotyping, p16/Ki67 dual staining, biomarkers, cytology, cervical cancer screening

## Abstract

**Background/Objectives**: The informative value of integrating high-risk human papillomavirus (HR-HPV) genotyping with cytology and p16/Ki67 dual-stain biomarker results, using limited and two types of extended genotyping assays, has not yet been evaluated. **Methods**: A total of 32,724 screening test results between 2015 and 2024 were included. Limited HPV genotyping was performed using the Abbott RealTime High Risk HPV assay. Extended genotyping was performed using two assays: the Alinity m HR HPV and BD Onclarity HPV Assay. Trends in age-specific, cytology-specific, and p16/Ki67-specific HR-HPV prevalence and distribution were observed, and differences between limited and extended genotyping were examined. **Results**: The overall HR-HPV positivity rate was 15.0%. HR-HPV prevalence was 13.9% in the limited genotyping group, 17.8% in in the Onclarity group 1, and 17.2% in the Alinity group 2, with a statistically significant difference in the proportions of positive/negative cases (*p* < 0.0001). No statistically significant difference was observed between extended genotyping groups (*p* = 0.706). In the Onclarity group: the highest p16/Ki67 positivity was observed for HPV 33/58 (100.0%) and HPV 31 (58.8%), while the lowest was for HPV 45 (18.2%), HPV 18 (25.0%) and HPV 59/56/66 (28.9%). In the Alinity group: the highest p16/Ki67 positivity was observed for HPV 16 (66.7%) and HPV 31/33/52/58 (58.8%). **Conclusions**: Based on ten years of HPV-based cervical cancer screening data, this study demonstrates that genotype-specific HR-HPV information obtained through extended genotyping provides clinically relevant risk stratification when interpreted together with cytology and p16/Ki67 dual-stain results. These findings support an integrated screening approach combining molecular HPV testing, cytology, and immunocytochemical biomarkers to improve risk-based triage in cervical cancer screening.

## Simple Summary

Cervical cancer screening increasingly relies on testing for high-risk human papillomavirus, but a positive result alone does not identify women at the highest risk of precancer or cancer. We analyzed ten years of screening data to evaluate the informative value of high-risk HPV genotyping across cytology categories using limited and extended genotyping approaches combined with p16/Ki67 dual-stain biomarker testing. Our findings indicate that extended genotyping provides clinically relevant information and that integrated interpretation of genotype-specific HR-HPV results with cytology and p16/Ki67 dual-stain may improve risk stratification and triage in cervical cancer screening.

## 1. Introduction

Primary testing for high-risk human papillomavirus is a globally recommended strategy for cervical cancer screening [1]. The transition from cytology-based to HPV-based screening has shifted clinical decision-making toward individualized risk estimation rather than test positivity alone. The 2019 Risk-Based Management Consensus Guidelines of the American Society of Colposcopy and Cervical Pathology (ASCCP) established a unified framework in which management is driven by the estimated risk of cervical intraepithelial neoplasia grade 3 or worse (CIN3+), integrating screening results across testing modalities and clinical history [2,3]. Within this framework, HPV genotype information plays a central role, as carcinogenic HPV types differ substantially in their oncogenic potential and natural history.

Initial genotype-informed management relied primarily on limited (partial) genotyping, most commonly distinguishing HPV 16 and HPV 18 from a pooled group of other carcinogenic types. Although clinically useful, subsequent analyses supporting the 2019 ASCCP guidelines demonstrated that limited genotyping provides only coarse risk stratification and fails to capture individual genotype-specific or grouped genotype–specific differences in CIN3+ risk [4]. More recent recommendations have therefore emphasized the clinical value of extended HPV genotyping, enabling risk assessment across individual genotype channels and biologically informed genotype groupings [5]. Currently, two FDA-approved assays providing extended genotyping are available for primary HPV screening, with the most recent approval in 2023 [6].

Advances in understanding the natural history of cervical HPV infection further support this approach, demonstrating that carcinogenic HPV genotypes follow distinct trajectories of persistence and progression, with variable risks of cervical precancer and cancer [7,8]. These insights favor a more information-rich interpretation of HPV test results, particularly when integrated with biomarkers of transforming infection.

The p16/Ki67 dual-stain (DS) test is an immunocytochemical method with the potential to improve detection of cervical precancerous lesions in HPV-based screening [9,10,11,12,13,14], and an equivalent triage strategy to limited HPV genotyping, colposcopy, and cytology [15]. Recently, triage options for HR-HPV-positive results were summarized in the study of the American Society of Cytopathology [16]. European multisocietal recommendations advocate biomarker-based, risk-adapted HPV screening, emphasizing the complementary roles of extended genotyping and p16/Ki67 dual-stain triage in HPV-positive populations [17,18].

The informative value of HR-HPV genotyping has not yet been evaluated by correlating limited and two types of extended genotyping with cytology and p16/Ki67 dual- stain results in a large screening cohort. Therefore, we conducted an integrated post hoc analysis of genotype-specific patterns, cytology, and p16/Ki67 biomarker status, and compared different HPV genotyping approaches in terms of their added value within current risk-based screening algorithms.

## 2. Materials and Methods

### 2.1. Study Population

**Figure 1:**
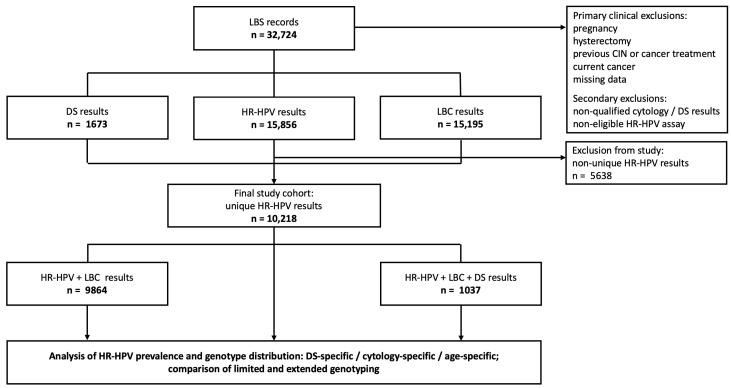
Profile of study population selection and construction of the analytical dataset. Cervical cancer screening records retrieved from the database were screened according to predefined primary clinical and secondary exclusion criteria. Cases with non-unique HR-HPV results were excluded to obtain the final cohort with unique HR-HPV results. The cohort was subsequently stratified according to the availability of HR-HPV testing, liquid-based cytology (LBC) and p16/Ki67 dual-stain (DS) results for further analyses of HR-HPV prevalence and distribution. Abbreviations: CIN, cervical intraepithelial neoplasia; DS, p16/Ki67 dual-stain; HR-HPV, high-risk types of human papillomavirus; LBC, liquid-based cytology; LBS, liquid-based screening.

In this study, 32,724 liquid-based cervical cancer screening (LBS) test results were retrospectively analyzed. The samples were obtained from opportunistic, privately funded cervical cancer screening conducted at the Corfamed Woman’s Health Center, a major private outpatient gynecological clinic located in an urban area in Poland. The analysis was based on virologic, cytologic, and immunocytochemical test results retrieved from the electronic database from August 2015 to March 2024. This study included the following initial screening tests: 15,856 for HR-HPV, 15,195 for LBC, and 1673 for DS. During the study period, two different screening models were implemented: primary cytology with reflex HR-HPV, and primary cotesting. The reflex HR-HPV test was performed in cases with atypical squamous cells of undetermined significance (ASC-US) or low-grade squamous intraepithelial lesions (LSIL) detected on primary cytology screening. DS testing was applied for all HR-HPV-positive results, and in cotesting-based screening models, in accordance with Polish national guidelines [18]. The LBS tests were performed on a diverse group of women aged 16–92 years, with a mean age of 41.02 years. The final study group consisted of 10,218 women. The selection process is shown in Figure 1. The screened population was predominantly composed of women from middle-to-upper socioeconomic strata, which reflects the private opportunistic screening conducted in an urban outpatient setting. Educational levels among participants were generally high; however, detailed quantitative data on socioeconomic status and educational level were not systematically collected in the screening database. Eligibility criteria were designed to enroll non-pregnant participants representative of a real-life screening population who had all investigated screening tests results available, including HR-HPV status, cytology, and DS, and who underwent standardized medical procedures: clinical management of abnormal screening test results with follow-up to ensure controlled disease ascertainment. The primary exclusion criteria included hysterectomy, current pregnancy, history of treatment for cervical intraepithelial lesions or cancer, current cancer, or missing data. Secondary exclusion criteria were: cytologic or DS reports interpreted by an unqualified or non-gynecological cytopathologist, and the use of HR-HPV molecular assays different from those specified in the enrollment criteria. Primary and secondary exclusion criteria were consistently applied across all enrollment protocols.

### 2.2. HR-HPV Molecular Testing with Limited or Extended Genotyping

HR-HPV detection was performed using one of three assays: the Abbott RealTime High Risk HPV assay (Abbott Molecular, Des Plaines, IL, USA), the BD Onclarity HPV Assay (Becton Dickinson, Franklin Lakes, NJ, USA), or Alinity m HR HPV Assay (Abbott Molecular, Des Plaines, IL, USA), all in accordance with the manufacturers’ instructions. The first assay detects 12 types of high-risk HPV DNA (31, 33, 35, 39, 45, 51, 52, 56, 58, 59, 66, and 68) in one group and identifies HPV 16 and HPV 18 independently (limited genotyping). The second assay individually detects HPV 16, HPV 18, HPV 45, HPV 31, HPV 52, and HPV 51 and reports the following genotype sets: HPV 33/58, HPV 35/39/68, and HPV 59/56/66. The Alinity HR HPV Assay detects HPV 16, HPV 18, and HPV 45 separately and classifies other HR-HPV types into two genotype sets: HPV 31/33/52/58 and HPV 35/39/51/56/59/66/68. Both the Onclarity and Alinity assays represent extended genotyping beyond HPV 16 and 18, enabling simultaneous detection of multiple viral genotypes. All screening samples were collected using a Cervex-Brush device (Rovers Medical Devices, Oss, The Netherlands; the catalog number: ROV 380100331).

### 2.3. Liquid-Based Cytology

SurePath liquid-based slides were prepared in external laboratories using an automated BD PrepStain Slide Processor (Becton Dickinson, Franklin Lakes, NJ, USA; RRID:SCR_026336) and BD Totalys SlidePrep (Becton Dickinson, Franklin Lakes, NJ, USA; RRID:SCR_026341) according to the manufacturers’ instructions. Residual cervical samples were stored in the laboratory for 1–3 months. A gynecological cytopathologist evaluated all cytology samples based on the Bethesda System 2014, with knowledge of the HR-HPV status of the samples (an informed cytology approach). Quality control procedures adhered to the standards of US laboratories accredited by the College of American Pathologists.

### 2.4. p16/Ki67 Dual Stain Testing

Dual immunocytochemical staining was performed using the CINtec PLUS Detection Kit, Roche (Basel, Switzerland), Cat# 10215348001 (RRID:AB_3675723). The kit contains a cocktail of two primary antibodies targeting human proteins: (1) a monoclonal mouse antibody (clone E6H4) directed against the cell cycle regulator p16^INK4a^ protein and (2) a recombinant rabbit antibody (clone 274-11AC3V1) directed against the proliferation marker Ki-67 protein. Immunocytochemical detection in cytological specimens was performed using an automated Ventana BenchMark XT system (Ventana Medical Systems, Tucson, AZ, USA; RRID:SCR_026335). The procedure was carried out as previously described [10,11,12] using residual cellular samples from the original SurePath vials after cytology and/or HPV testing. Immunoprofile assessment was conducted by an experienced gynecological cytopathologist who was blinded to the cytology and HR-HPV test results.

### 2.5. Informative HR-HPV Genotyping Approach

The term informative HR-HPV genotyping (informative HPV) refers to genotype-specific HR-HPV results used to support risk-stratified clinical assessment, as applied in this study in conjunction with cytology and p16/Ki67 dual-stain. This approach enables an integrated evaluation of genotype-specific HR-HPV patterns across cytological categories and biomarker status within precision cervical cancer prevention.

### 2.6. Statistical Analysis

The distribution of HR-HPV was described using prevalence and type-specific HR-HPV positivity rates (%). Prevalence was defined as the proportion of positive test results. Statistical analyses were conducted to compare the distribution of positive and negative results across different test groups. The chi-square test (χ^2^) was used to determine the overall associations, followed by pairwise comparisons using the chi-square test with Bonferroni correction. The McNemartest was applied to assess the significance of the differences between DS-positive and LBC-positive (ASC-US+) results in HR-HPV-positive cases. A *p* value < 0.05 was considered statistically significant. This study was a retrospective analysis, and the sample size was determined by the availability of all eligible data to maximize the dataset for analysis. All statistical analyses were conducted using PQStat Statistical Calculation Software—version 2015 (RRID:SCR_026338).

## 3. Results

### 3.1. Overall HR-HPV Prevalence and Trends Observed

**Figure 2:**
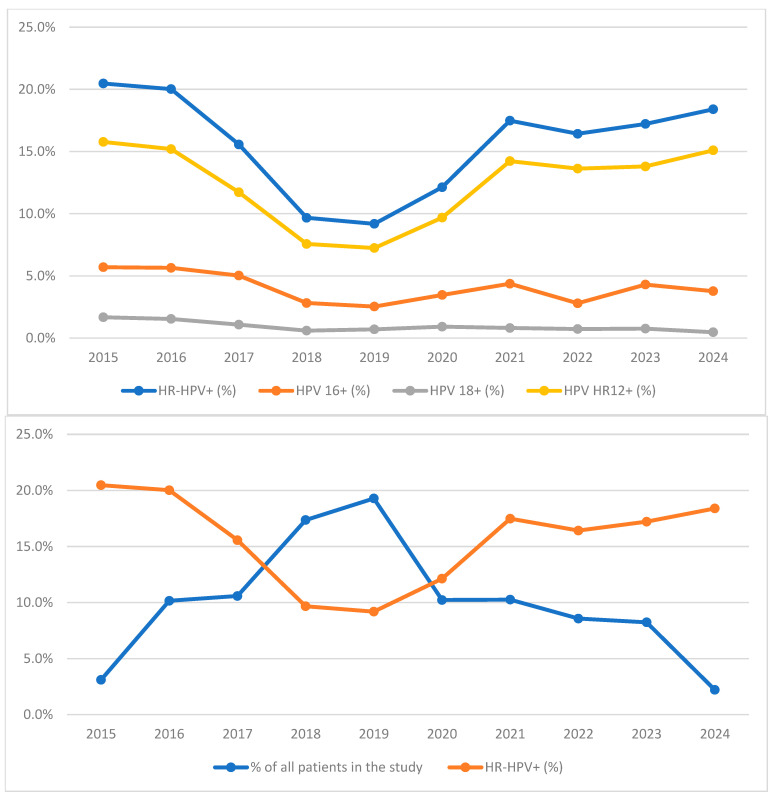
Annual HR-HPV prevalence and screening activity during 10-year study period. (On the (**top**)) Annual prevalence of HR-HPV infection in the screened population; (on the (**bottom**)) relationship between HR-HPV prevalence and the annual number of HR-HPV tests performed. Abbreviations: HR-HPV, high-risk types of human papillomavirus; HR-HPV+, 14 high-risk types of human papillomavirus positive results; HPV 16+, human papillomavirus type 16 positive results; HPV 18+, human papillomavirus type 18 positive results; HPV HR12+, human papillomavirus 12 high-risk types other than types 16 and 18 positive results.

In the final study group consisting of 10,218 women, 15.0% (1528/10,218) were HR-HPV-positive. Among HR-HPV-positive cases, 4.2% (427/10,218) were HPV 16-positive, 0.9% (96/10,218) were HPV 18-positive, and 11.6% (1189/10,218) were HPV HR12-positive (non-16/non-18 HR-HPV types). The annual HR-HPV prevalence over the 10-year study period is shown in Figure 2. During the first four years of our study, the overall HR-HPV prevalence, comprising all types of molecular testing performed, showed a gradual decrease. However, an opposite trend was observed, with a slow increase from that point until the end of the study period. The observed decrease might be associated with the larger sample size recorded in 2018–2019.

### 3.2. Limited vs. Extended Genotyping

**Figure 3:**
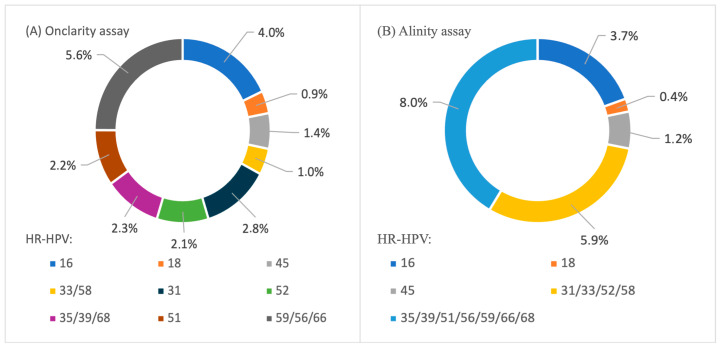
Distribution of individual and grouped high-risk HPV genotypes detected using extended genotyping assays. The left chart shows results obtained with the Onclarity assay and the right chart shows results obtained with the Alinity assay. Percentages represent the proportion of each genotype or genotype group among HR-HPV-positive cases within the respective assay group. Grouped genotypes correspond to assay-specific reporting categories used in extended HPV genotyping platforms. Abbreviations: HR-HPV, high-risk types of human papillomavirus.

HR-HPV positivity using limited genotyping was observed in 1009 cases, with HPV HR12 (10.6%) and HPV 16 (4.3%) being the most frequently detected genotypes. Molecular testing with extended genotyping identified HR-HPV positivity in 519 cases, including 243 cases using the Onclarity assay and 276 cases with the Alinity assay. This subset enabled a more detailed analysis of HR-HPV genotype distribution (Figure 3). In the Onclarity group, the most prevalent genotypes or genotype groups were HPV 59/56/66 (5.6%), HPV 16 (4.0%), and HPV 31 (2.8%). The least frequent were HPV 33/58 (1.0%) and HPV 18 (0.9%). Among women tested with Alinity, the genotype group HPV 35/39/51/56/59/66/68 (8.0%) was the most frequently detected, whereas HPV 18 was the least frequently detected genotype (0.4%). The combined prevalence of HPV 33/58 (1.0%) and individual genotypes HPV 31 (2.8%) and HPV 52 (2.1%) in the Onclarity group was 5.9%, whereas the corresponding genotype group HPV 31/33/52/58 in the Alinity group showed an identical prevalence of 5.9%. Similarly, HPV 35/39/68 (2.3%), HPV 59/56/66 (5.6%), and HPV 51 (2.2%) in the Onclarity group together accounted for 10.1% of women, whereas the corresponding genotype group HPV 35/39/51/56/59/66/68 in the Alinity group was detected in 8.0% of women.

The overall HR-HPV prevalence was 13.9% for the Abbott assay, 17.8% for the Onclarity assay, and 17.2% for the Alinity assay; due to overlapping co-infections, genotype categories in Figure 3 are not mutually exclusive. A statistically significant difference in the proportions of positive and negative cases was observed among the three assay groups (*p* < 0.0001). The observed variation in HR-HPV prevalence between the Abbott group (7250 participants) and the two extended genotyping groups (2968 participants) may partly reflect differences in sample size as well as the sequential implementation of assays during the study period. This interpretation is consistent with the lower HR-HPV prevalence recorded during the 2018–2019 period, when the study cohort reached its largest size. Pairwise comparisons demonstrated statistically significant differences between the Abbott and Onclarity groups (*p* = 0.00022), and between the Abbott and Alinity groups (*p* = 0.00083), with *p*-values well below the adjusted significance threshold (0.0167). In contrast, no statistically significant difference was observed between the Onclarity and Alinity groups (*p* = 0.706).

### 3.3. Age-Specific HR-HPV Prevalence and Distribution

**Figure 4:**
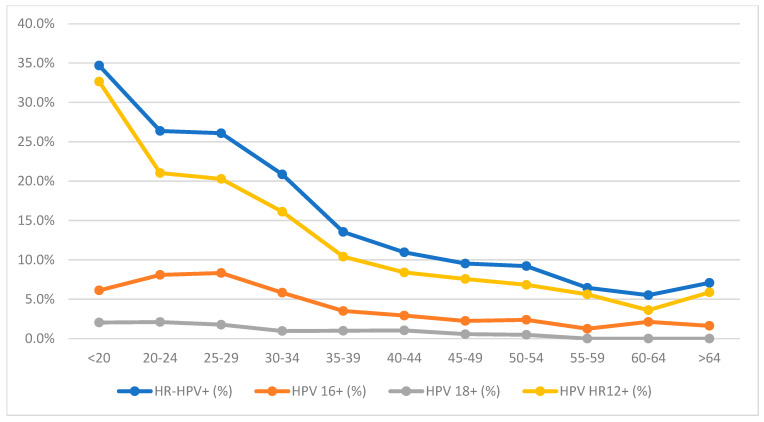
The age-specific prevalence of HR-HPV infection. Abbreviations: HR-HPV, high-risk types of human papillomavirus; HR-HPV+, 14 high-risk types of human papillomavirus positive results; HPV 16+, human papillomavirus type 16 positive results; HPV 18+, human papillomavirus type 18 positive results; HPV HR12+, human papillomavirus 12 high-risk types other than types 16 and 18 positive results.

The study population was divided into the following age groups: <20, 20–24, 25–29, 30–34, 35–39, 40–44, 45–49, 50–54, 55–59, 60–64, and >64 years. The largest subgroup consisted of women aged 35–39 years (*N* = 1912), followed closely by those aged 30–34 (*N* = 1663) and 40–44 years (*N* = 1643). The smallest subgroup was noted in women aged <20 years (*N* = 49). Differences in HR-HPV prevalence and genotype distribution were observed across age groups. The highest HR-HPV prevalence was recorded among women aged <20, 20–24, and 25–29 years (34.7%, 26.4%, and 26.1%, respectively). A decreasing trend in HR-HPV prevalence was observed with increasing age, with the exception of the oldest age group, where a slight increase was noted. Patterns of genotype distribution did not fully correspond to overall prevalence trends. HPV 16 was most prevalent among women aged 25–29 (8.3%) and 20–24 years (8.1%) and least frequent in the >64 age group (1.6%) and 55–59 (1.2%) age groups. HPV HR12 was most prevalent in the <20 age group (32.7%), and least common among women aged 60–64 years (3.6%). Detailed results are presented in Figure 4.

### 3.4. Cytology-Specific HR-HPV Prevalence and Distribution

In the final study group, LBC results were available for 9864 women, of whom 12.2% had ASC-US+ cytology. The highest HR-HPV prevalence was observed among women with HSIL (96.7%), ASC-H (85.1%), and LSIL (80.0%), whereas the lowest prevalence was noted in NILM cases (7.6%). Among women with NILM, ASC-US, LSIL, and ASC-H cytology results, HPV HR12 was the most frequently detected genotype, whereas in HSIL cases HPV 16 was the predominant genotype. Detailed results are shown in Table 1, excluding AGC, adenocarcinoma, and unsatisfactory cytology results (*N* = 45).

In HPV 16-positive cases, 33.2% of women had NILM cytology, 29.6% ASC-US, 20.6% LSIL, 10.1% HSIL, and 5.3% ASC-H. Among HPV 18-positive women, 37.8% had NILM cytology, 26.7% ASC-US, 23.3% LSIL, 5.6% HSIL, and 3.3% ASC-H. Among women positive for HPV HR12, cytology results were distributed as follows: 47.3% NILM, 24.1% ASC-US, 23.6% LSIL, 2.6% ASC-H, and 1.9% HSIL.

**Figure 5:**
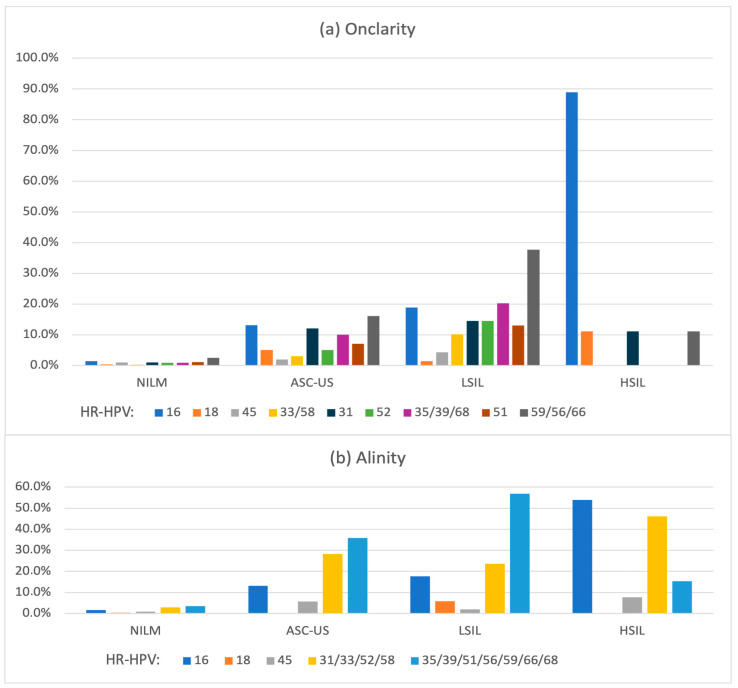
Cytology-specific distribution of HR-HPV genotypes in women tested with the extended genotyping assays: Onclarity (**a**) and Alinity (**b**). Bars represent the percentage of HR-HPV-positive cases for individual genotypes or genotype groups within each cytological category. Abbreviations: HR-HPV, high-risk types of human papillomavirus; NILM, negative for intraepithelial lesion or malignancy; ASC-US, atypical squamous cells of undetermined significance; LSIL, low-grade squamous intraepithelial lesion; HSIL, high-grade squamous intraepithelial lesion.

In the Onclarity group, HPV 59/56/66 was the most prevalent genotype group among women with NILM, ASC-US, and LSIL cytology results, whereas HPV 16 was the most frequently detected in HSIL cases. Similar patterns were observed in the Alinity group, where the HPV 35/39/51/56/59/66/68 genotype group was the most prevalent in NILM, ASC-US, and LSIL cases, while HPV 16 was predominant in HSIL cytology. Detailed data for HR-HPV-positive cases are presented in Figure 5. Data for the ASC-H category was excluded due to the relatively small sample size in the Onclarity and Alinity study groups.

### 3.5. DS-Specific HR-HPV Prevalence and Distribution

**Figure 6:**
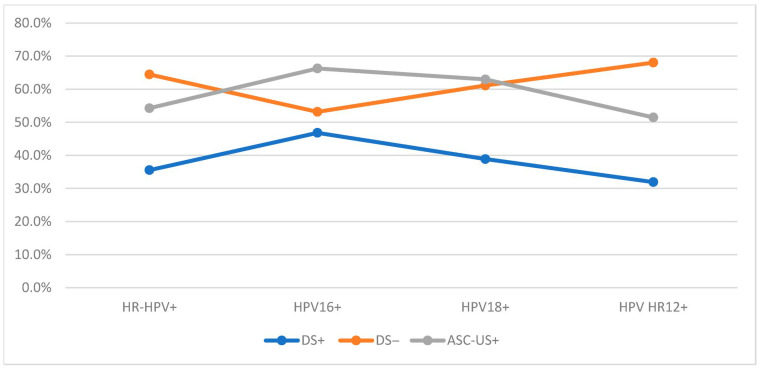
The comparison of DS and LBC results in HR-HPV-positive patients. Abbreviations: HR-HPV, high-risk types of human papillomavirus; HR-HPV+, 14 high-risk types of human papillomavirus positive results; HPV 16+, human papillomavirus type 16 positive results; HPV 18+, human papillomavirus type 18 positive results; HPV HR12+, human papillomavirus 12 high-risk types other than types 16 and 18 positive results; DS, p16/Ki67 dual-stain testing; ASC-US+, atypical squamous cells of undetermined significance or worse; +, positive; −, negative.

In the final study group, 1037 women underwent DS testing with available results, of whom 844 (81.4%) were HR-HPV-positive. Within this subgroup, 35.5% of women were DS-positive and 64.5% were DS-negative. Among HPV 16-positive women, 46.8% were DS-positive and 53.2% were DS-negative. Among HPV 18-positive women, 38.9% were DS-positive and 61.1% were DS-negative, while among HPV HR12-positive women, 31.9% were DS-positive and 68.1% DS-negative. The proportions of ASC-US+ cytology in the corresponding HR-HPV-positive subgroups (Figure 6) were significantly higher: 66.3% for HPV 16-positive cases (*p* < 0.0001), 63.0% for HPV 18-positive cases (*p* = 0.0028), and 51.5% for HPV HR12-positive cases (*p* < 0.0001).

**Figure 7:**
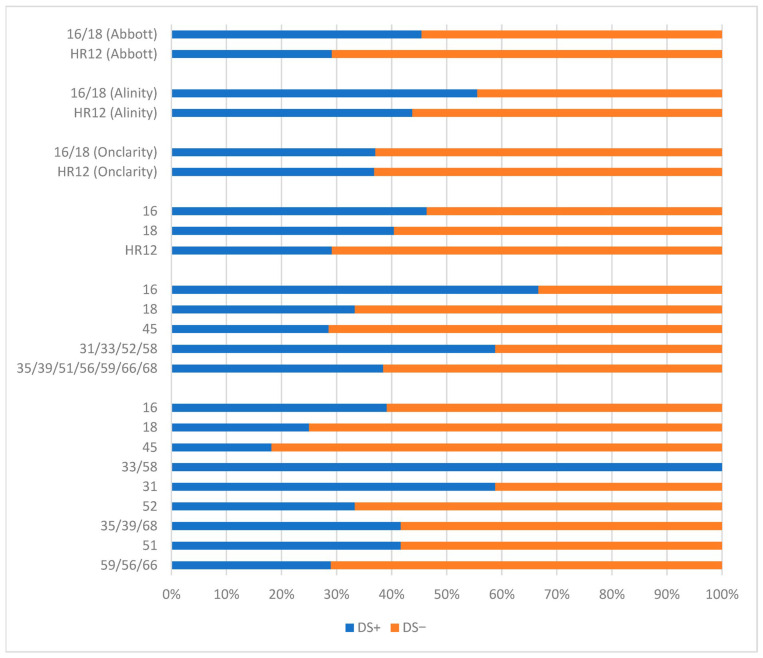
The DS results in HR-HPV-positive patients using the Abbott assay (first from the top and third from the bottom), Alinity assay (second from the top and second from the bottom) and Onclarity assay (third from the top and first from the bottom). Abbreviations: DS, p16/Ki67 dual-stain testing; +, positive; −, negative; 16/18, human papillomavirus types 16 and/or 18; HR12, human papillomavirus 12 high-risk types other than types 16 and 18.

In the Onclarity group, 114 HR-HPV-positive women underwent DS testing, while in the Alinity group, 52 women met the same inclusion criteria. In the Onclarity group, the highest DS positivity was observed for HPV 33/58 (100.0%) and HPV 31 (58.8%), whereas the lowest positivity was noted for HPV 45 (18.2%), HPV 18 (25.0%), and HPV 59/56/66 (28.9%). In the Alinity group, the highest DS positivity was observed for HPV 16 (66.7%) and HPV genotype group 31/33/52/58 (58.8%), with the lowest positivity detected in the same genotype groupsas in the Onclarity assay. Detailed data for extended genotyping results are presented in Figure 7.

## 4. Discussion

This is the first study to determine the informative value of HR-HPV genotyping across cytology in combination with the p16/Ki67 dual-stain biomarker, using limited genotyping for HPV 16 and/or 18 (the Abbott study group) and two types of extended genotyping identifying different individual HPV genotypes or genotype sets (the Alinity and Onclarity study groups). Our analysis demonstrates that genotype-specific HR-HPV distribution differs substantially across the investigated screening groups, and that extended genotyping provides additional information that may refine risk stratification within HPV-positive women in HPV-based cervical cancer screening. The prevalence of HR-HPV differed significantly between the Abbott and Onclarity study groups (*p* = 0.00022) and between the Abbott and Alinity study groups (*p* = 0.00083), whereas no statistically significant difference was observed between the two extended genotyping assays (*p* = 0.706).

The overall HR-HPV prevalence in our study population was 15.0%. HPV 16 was detected in 4.2% of women, HPV 18 in 0.9%, and HPV HR12 in 11.6%. In the Abbott group, the most common genotypes were HPV HR12 (10.6%) and HPV 16 (4.3%). In the Onclarity group, the most prevalent genotypes or genotype sets were HPV 59/56/66 (5.6%), HPV 16 (4.0%), and HPV 31 (2.8%). In the Alinity group, the most frequently detected genotype sets were HPV 35/39/51/56/59/66/68 (8.0%), HPV 31/33/52/58 (5.9%) and HPV 16 (3.7%), with the highest prevalence observed in younger women (26.1–34.7%). Consistently, HPV 16 predominated in the younger age groups (8.1–8.3%). HR-HPV positivity was highest in HSIL cases (96.7%), and lowest in NILM (7.6%). Among the NILM, ASC-US, LSIL, and ASC-H cytology categories, HPV HR12 was the most frequently detected. In the Onclarity group, the genotype set HPV 59/56/66 predominated in these cytological categories, whereas HPV 16 was the predominant genotype in HSIL cases. Among HR-HPV-positive women, 35.5% were DS-positive and 64.5% were DS-negative, with DS positivity varying by genotype: 46.8% for HPV 16, 38.9% for HPV 18, and 31.9% for HPV HR12. In contrast, the proportion of ASC-US+ cytology in the corresponding subgroups was significantly higher: 66.3%, 63.0%, and 51.5%, respectively. One clinically relevant observation concerns the difference between women who are HPV HR12-positive with a positive DS result (31.9% for HR12-positive and DS-positive) and those with abnormal cytology (51.5% for HR12-positive and ASC-US+), reflecting different triage approaches used in HPV-based screening. This finding suggests that integrating genotype-specific HR-HPV information with biomarker-based triage may help refine risk stratification among women positive for HPV HR12. According to current guidelines recommending limited HPV 16/18 genotyping, women positive for HPV HR12 are typically referred directly for colposcopy without triage [19,20,21,22].

The overall HR-HPV prevalence observed in our study was consistent with estimates reported in other European populations [23,24]. Galati et al. described a comparable prevalence, highlighting the widespread nature of HR-HPV infections across Europe (14.4% vs. 15.0% in our study) [23]. However, our findings were slightly lower than those reported in Northern European countries such as Sweden and Denmark (15.0% vs. 18.1%) [24], which may reflect differences in sexual behavior, screening practices, and population demographics. Age-specific HR-HPV prevalence patterns in our cohort were similar to those observed in other European studies, including Nordic countries and Spain. In women younger than 30 years, the prevalence was lower than that reported in Nordic populations, whereas in women younger than 40 years it was higher than that reported in Spain. In older age groups, the prevalence estimates were largely comparable [24,25]. Our findings aligned with Polish data [26], although both the overall HR-HPV prevalence and HPV 16 prevalence were slightly lower in our cohort, with the most notable differences observed in the 35–44-year age range. Notably, HR-HPV prevalence in our cohort was substantially lower than that reported in a Chinese study [27] and in another Polish dataset reported by Glinska et al. [28] (39% vs. 15.0%), likely reflecting the study population characteristics. Compared with data from the United States, the overall HR-HPV prevalence in our study was slightly higher (15.0% vs. 9.8%/10.5%) [29,30]. Age-specific prevalence estimates were broadly similar to those reported by Trama et al. [29], although differences of approximately 10% were observed in three age groups (26–30, 31–35, and 36–40 years—15.2%/10.8%/6.4%—vs. 25–29, 30–34, and 35–39 years—26.1%/20.9%/13.5%—in our study). In the remaining age groups, differences were minor and likely reflecting the larger proportion of younger women in our cohort. Similarly, age-specific HR-HPV prevalence was slightly higher than that reported by Wright et al., with the largest differences observed in the 30–39 age groups, although these differences remained below 10% [30].

Regarding HR-HPV genotype distribution (the most detailed genotyping in our study was obtained in the Onclarity group using extended genotyping), HPV 16 was the most prevalent individual genotype, consistent with global data identifying this genotype as the most common and oncogenic (4.0% vs. 5.9%, 4.9%, 5.6%, 3.4%, and 3.5%) [23,24,31,32]. Our data also showed that the HPV 59/56/66 genotype set accounted for a substantial proportion of infections. The prevalence of other HR-HPV genotypes was comparable to that reported in European studies, including for HPV 31 (2.8% vs. 2.8%/2.9%) [23,24], HPV 51 (2.2% vs. 2.1%) [23], HPV 52 (2.1% vs. 2.8%) [24], and HPV 45 (1.4% vs. 0.8%) [23]. Compared with data from the United States [29], the prevalence of HPV 16 (1.2% vs. 4.0%), HPV 31 (0.5% vs. 2.8%), and HPV 59/56/66 (2.0% vs. 5.6%) was higher in our study, whereas the prevalence of HPV 18, HPV 45, HPV 51, HPV 52, HPV 33/58 and HPV 35/39/68 was comparable. In a recent Chinese study, HPV 52, HPV 33/58 and HPV 35/39/68 were more prevalent than in our cohort, whereas HPV 45 and HPV 31 were less common [33]. The prevalence of HPV 16, HPV 18, HPV 51, and HPV 59/56/66 was similar. These differences could suggest regional variation in genotype distribution, potentially influenced by differences in the local HPV epidemiology, host genetic factors, or environmental factors. A direct comparison of individual viral genotypes 33, 35, 39, 56, 58, 59, 66, and 68 was not possible because the Onclarity assay reports these types in grouped genotype channels.

The cytology-specific HR-HPV prevalence observed in our study was similar to that reported by Wang et al. [27]. Compared with data from the United States, our estimates were relatively higher across cytological categories, with the closest agreement noted in NILM cytology [29]. Furthermore, our cytology-specific results were higher for HPV HR12-positive women with NILM, ASC-US, LSIL and ASC-H cytology than those reported by Wentzensen et al. [34]. In contrast, the results for HSIL cytology were very similar between studies (36.0% vs. 37.9% in our study). The overall HR-HPV prevalence in ASC-H cases was slightly higher in our study than reported by Sun et al. (85.1% vs. 78.5%). In this comparison, HPV 16 (44.7% vs. 33.8%) and HPV 18 (6.4% vs. 4.7%) were more frequent in our cohort, whereas HPV HR12, excluding cases with HPV 16 and HPV 18 co-infection, was slightly less prevalent (36.2% vs. 39.1%) [35].

The percentage of DS-positive cases among HPV HR12-positive women in our study was lower than reported by Wright et al. (31.9% vs. 47.5%) [36]. The observed 100% DS positivity for the HPV 33/58 and for ASC-H cases in the extended genotyping groups should be interpreted with caution due to the small number of cases in these subgroups.

Our study highlights the importance of ongoing surveillance of HR-HPV genotype distribution in the context of biomarker-based cervical cancer screening strategies. The implementation of HPV vaccination programs is expected to modify the prevalence of vaccine-covered genotypes and may lead to a relative increase in non-vaccine high-risk HPV genotypes. In this setting, informative HR-HPV genotyping integrated with cytology and p16/Ki67 dual-stain testing may provide added clinical value by improving risk stratification in HPV-positive women. Continuous monitoring of genotype prevalence will therefore be important for assessing the long-term impact of vaccination and for guiding future vaccine development strategies. This study has several notable strengths. It includes one of the largest real-world datasets combining HR-HPV genotyping, LBC, and p16/Ki67 dual-stain results, providing robust evidence for informed cervical cancer screening guidelines. The broad age range of participants reflects opportunistic screening conditions and enhances the generalizability of the findings. All cytological and immunocytochemical evaluations were performed by a qualified gynecological cytopathologist, ensuring consistency and standardized interpretation across all screening modalities.

Potential limitations include the single-center design, the privately funded setting, the limited geographic coverage, and a relatively homogeneous study population. Detailed quantitative data on socioeconomic status and educational level were not systematically collected. As the screening was conducted in a private urban clinic, the study population likely represents women with relatively higher socioeconomic and educational backgrounds, which may introduce a degree of selection bias. However, these characteristics reflect the routine clinical context of opportunistic cervical cancer screening in private healthcare settings. Another consideration concerns the observed differences in HR-HPV prevalence between assays using limited genotyping (Abbott) and extended genotyping (Onclarity and Alinity). During the study period, molecular assays were introduced and replaced sequentially as part of routine laboratory practice or following decisions made by the gynecological center, and were therefore not implemented concurrently. Consequently, no overlapping reference periods were available for direct comparison. Importantly, the screened population remained relatively homogeneous throughout the study period, which may mitigate the possibility that the observed differences reflect major population shifts rather than assay-related factors. The study was not designed as a controlled head-to-head comparison of assays but rather reflects real-world cervical cancer screening practice in a large cohort where testing platforms were introduced sequentially in response to evolving laboratory practice and technological developments.

## 5. Conclusions

In conclusion, the prevalence and genotype distribution of HR-HPV among women in Poland are broadly consistent with patterns reported in other European countries and the United States, with some regional distinctions. Based on a 10-year HPV-based screening dataset, our findings highlight the informative value of HR-HPV genotyping in combination with cytology and p16/Ki67 dual-stain biomarkers. This integrated approach may improve risk stratification and support more informed clinical decision-making in HPV-based cervical cancer screening. Furthermore, it may facilitate the implementation of new diagnostic technologies within contemporary screening programs. Continued surveillance of genotype distribution, public health education, vaccination efforts, and further research on genotype-specific risk stratification remain essential to optimize national screening strategies and strengthen the prevention of cervical precancer and cancer.

## Figures and Tables

**Table 1:** The cytology-specific HR-HPV distribution.

	NILM (*n* = 8642) No (%^1^/%^2^)	ASC-US (*n* = 664) No (%^1^/%^2^)	LSIL (*n* = 406) No (%^1^/%^2^)	ASC-H (*n* = 47) No (%^1^/%^2^)	HSIL (*n* = 60) No (%^1^/%^2^)
HPV 16+	132 (20.1/1.5)	118 (33.1/17.8)	82 (25.2/20.2)	21 (52.5/44.7)	40 (69.0/66.7)
HPV 18+	34 (5.2/0.4)	24 (6.7/3.6)	21 (6.5/5.2)	3 (7.5/6.4)	5 (8.6/8.3)
HPV HR12+	536 (81.5/6.2)	267 (74.8/40.2)	273 (84.0/67.2)	30 (75.0/63.8)	22 (37.9/36.7)
HR-HPV+	658 (100/7.6)	357 (100/53.8)	325 (100/80.0)	40 (100/85.1)	58 (100/96.7)

HR-HPV, high-risk types of human papillomavirus; HPV 16+, human papillomavirus type 16 positive results; HPV 18+, human papillomavirus type 18 positive results; HPV HR12+, human papillomavirus 12 high-risk types other than types 16 and 18 positive results; HR-HPV+, 14 high-risk types of human papillomavirus positive results; NILM, negative for intraepithelial lesion or malignancy; ASC-US, atypical squamous cells of undetermined significance; LSIL, low-grade squamous intraepithelial lesion; ASC-H, atypical squamous cells-cannot exclude HSIL; HSIL, high-grade squamous intraepithelial lesion; %^1^ Percentage of HR-HPV-positive results within each LBC category; %^2^ Percentage of total results within each LBC category.

## Data Availability

The original contributions presented in this study are included in the article. Further inquiries can be directed to the corresponding author.

## Abbreviations

The following abbreviations are used in this manuscript:

HR-HPV	High-risk human papillomavirus
ASCCP	American Society for Colposcopy and Cervical Pathology
CIN3+	Cervical intraepithelial neoplasia grade 3 or more severe diagnoses
HPV	Human papillomavirus
FDA	Food and Drug Administration
DS	p16/Ki67 dual staining
LBS	Liquid-based screening
ASC-US	Atypical squamous cells of undetermined significance
LSIL	Low-grade squamous intraepithelial lesions
RRID	Research Resource Identifiers
ASC-US+	Atypical squamous cells of undetermined significance or worse cytologic result
HR-HPV+	14 high-risk types of human papillomavirus positive results
HPV 16+	Human papillomavirus type 16 positive results
HPV 18+	Human papillomavirus type 18 positive results
HPVHR12+	Human papillomavirus 12 high-risk types other than types 16 and 18 positive results
LBC	Liquid-based cytology
NILM	Negative for intraepithelial lesion or malignancy
ASC-H	Atypical squamous cells-cannot exclude HSIL
HSIL	High-grade squamous intraepithelial lesion
AGC	Atypical glandular cells
